# Migration trachéale d'une canule de trachéotomie: complication exceptionnelle

**DOI:** 10.11604/pamj.2014.18.41.4201

**Published:** 2014-05-12

**Authors:** Chakib Chouikh, Amine El Moqaddem, Anas Benmakhlouf, Saad Naanaa, Alae El Koraichi, Salma El Kettani, Ali Jahidi

**Affiliations:** 1 Service de Réanimation Pédiatrique, Hôpital d'enfants Rabat, Rabat, Maroc; 2 Service ORL et CCF, Hôpital Militaire d'Instruction Mohammed V Rabat, Rabat, Maroc

**Keywords:** Trachéotomie canule, Krishaber, trachée, migration, tracheostomy cannula, Krishaber, trachea, migration

## Abstract

La trachéotomie est un geste chirurgical de survie largement pratiqué dans les services des urgences et de réanimation. En fonction de l'indication de sa réalisation, elle peut être transitoire ou définitive. Dans ce dernier cas le port d'une canule de trachéotomie de manière prolongée peut exposer à certaines complications qui peuvent s'avérer graves. Nous présentons un cas très rare d'un enfant présentant un syndrome de Guillain Barré, trachéotomisé depuis 4 ans suite à une sténose trachéale par intubation prolongée et portant une canule de trachéotomie métallique de type KRISHABER qui s'est présenté aux urgences dans un tableau de détresse respiratoire suite à la migration trachéale de sa canule. La trachéotomie est l'ouverture à la peau de la trachée cervicale, et à la mise d'une canule qui a pour but de permettre la respiration en court-circuitant les voies aériennes supérieures. De réalisation simple et codifiée le plus souvent, elle présente des risques de complications post opératoires notamment tardives. Les plus décrites sont les granulomes, les sténoses trachéales, les infections, et les fistules. La migration trachéale de la canule de trachéotomie reste exceptionnelle. Elle résulte d'un mauvais entretien qui fragilise la canule et doit être prise en charge en urgence. La trachéotomie définitive nécessite une surveillance régulière, un entretien et des soins de canules rigoureux pour éviter la survenue de complications qui peuvent être graves. Chez l'enfant, l'utilisation de canules souples en PVC ou en silicone doit être préférée aux canules métalliques.

## Introduction

**Figure 1:**
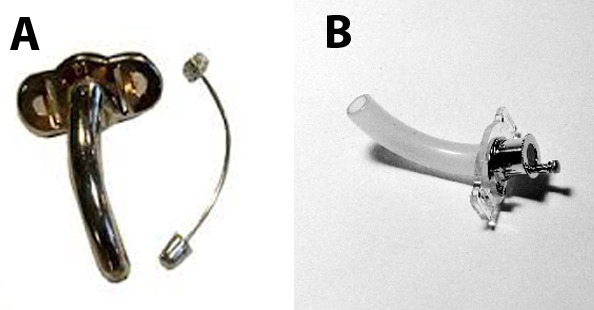
Modèles de canules de KRISHABER; A)métallique; B) plastic

La trachéotomie consiste en l'ouverture antérieure de la trachée cervicale à travers une incision cutanée et à la mise en place d'une canule qui a pour but de permettre la respiration en court-circuitant les voies aériennes sus-glottiques. La canule de trachéotomie ou canule de KRISHABER a été mise au point en 1850 par Maurice KRISHABER. Il s'agit d'un tube courbe métallique (Figure 1-A) ou en plastic (Figure 1-B), avec un angle de 110 à 130 degrés dont une extrémité pénètre dans la trachée à travers l'orifice de la trachéotomie, et l'autre extrémité est extérieure apparente permettant sa fixation. Dans le cas de trachéotomie définitive, plusieurs complications ont été rapportées dans la littérature, les plus fréquentes étant les granulomes, l'infection et la sténose [1]. Nous rapportons à travers notre observation un cas exceptionnel et très grave de migration trachéale liée au port prolongé de canule de trachéotomie.

## Patient et observation

**Figure 2:**
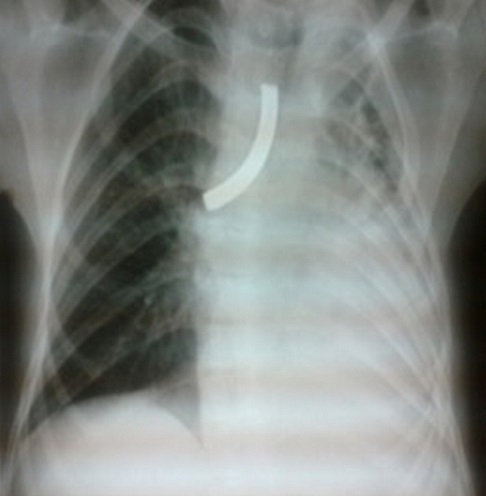
Radiographie thoracique montrant la canule en intra trachéal se dirigeant vers la bronche souche droite

Il s'agit d'un enfant de 15 ans hospitalisé en 2008 en réanimation pédiatrique pour un syndrome de Guillain barré, dont la prise en charge a nécessité une intubation prolongée suivie d'une trachéotomie définitive pour une sténose trachéale. Le patient est sorti après 6 mois portant une canule de KRISHABER de type métallique. 4 ans plus tard, il est réadmis aux urgences dans un tableau de détresse respiratoire en rapportant la migration trachéale de sa canule. L'examen trouvait un enfant conscient stable sur le plan hémodynamique fortement dyspnéique avec des signes de lutte. La radiographie thoracique (Figure 2) a montré la présence de la canule en regard de la carène avec atélectasie pulmonaire gauche. En effet, elle s'est introduite dans la bronche souche droite et permettait la ventilation pulmonaire de ce côté mais obstruait totalement la bronche souche gauche. L'enfant a été transféré en urgence au bloc opératoire O.R.L où il a bénéficié d'une extraction de la canule sous endoscopie avec réfection de la trachéotomie. Les suites opératoires étaient simples avec amélioration clinique et radiologique, le patient est sorti a domicile avec une canule en plastique après avoir expliqué aux parents l'importance des sois et de l'entretien de la canule.

## Discussion

La trachéotomie est l'ouverture à la peau de la trachée cervicale suivie de la mise en Place d'une canule. Destinée à réaliser un court-circuit des voies aériennes Supérieures (VAS) [1], elle se réalise d'une manière chirurgicale ou percutanée, sous anesthésie locale ou générale.

Ses indications sont multiples et comptent essentiellement les détresses respiratoires dues aux obstacles des VAS, aux traumatismes trachéaux et aux tumeurs pharyngo-laryngées, mais aussi les intubations difficiles, la ventilation assistée au long cours et l'insuffisance respiratoire chronique.

Chez l'enfant les causes les plus fréquentes sont la papillomatose laryngée, la laryngite aigue et la sténose laryngo-trachéale [2], cette dernière peut être iatrogène, ce qui est le cas de notre patient.

La trachéotomie est considérée actuellement comme un geste thérapeutique de survie avec une technique simple, mais greffée parfois de multiples complications, surtout si elle est définitive.

Depuis la pratique de cette technique, plusieurs types de canule ont été utilisées (rigides, souples, fenêtrées métalliques en plastique ou en silicone), le choix dépend de l'indication et de la morphologie de chaque patient, chez l'enfant on préfère les canules souples, mieux tolérées par la muqueuse trachéale [3], sans chemise interne, ce qui permet des soins faciles. Notre enfant portait une canule rigide en argent type KRISHABER avec trois parties, une chemise externe en contact avec la trachée munie à son extrémité d'une collerette sur laquelle on adapte une bande permettant la fixation de la canule, une chemise interne et un mandrin permettant d'introduire la chemise externe dans la trachée grâce à son bout arrondi et non traumatisant.

Les complications tardives liées au port de la canule les plus rapportées dans la littérature sont les granulomes periorificiels, les sténoses trachéales, les infections, et les fistules (trachéo-bronchique, trachéo-cutanées et trachéo-innominées) qui sont très rares [4, 5]. La migration intra trachéale de la canule reste exceptionnelle. Elle est due dans notre cas à l'usure et la fragilisation de la collerette qui a fini pas se rompre et se désolidariser du corps de la canule qui a ainsi glissé le long de la lumière trachéale. La cause vraisemblablement de cet incident est le non respect des impératifs d'entretien de la canule souvent négligés par les patients.

Ces complications peuvent être évitées par des soins réguliers de l'orifice et de la canule de trachéotomie, une surveillance rigoureuse à travers des consultations régulières. La sensibilisation du patient et ses proches sur l'importance des soins, le nettoyage des canules, les aspirations, et des conduites face aux incidents permettant au minimum le transfert des patients a l'hôpital est primordiale.

Chez l'enfant l'utilisation de canules souples en silicone ou PVC est préférable car moins traumatisantes que les canules métalliques [2, 3]. Le petit diamètre de la trachée impose le mise en place de canule sans chemise interne ce qui impose des soins et une surveillance encore plus réguliers.

## Conclusion

Quelque soit l'indication, la trachéotomie est un geste de survie dont l'utilité et l'efficacité sont certaines. La maîtrise de la technique, le bon choix du matériel, la parfaite connaissance des rapports anatomiques de la trachée, la surveillance rigoureuse et les soins postopératoires représentent les principales conditions pour minimiser les risques de survenue des complications, parmi lesquelles la migration trachéale de la canule de Krishaber reste exceptionnelle.
